# PET measured hypoxia and MRI parameters in re-irradiated head and neck squamous cell carcinomas: findings of a prospective pilot study

**DOI:** 10.12688/f1000research.27303.2

**Published:** 2021-03-16

**Authors:** Julian Rogasch, Marcus Beck, Carmen Stromberger, Frank Hofheinz, Pirus Ghadjar, Peter Wust, Volker Budach, Holger Amthauer, Ingeborg Tinhofer, Christian Furth, Thula C. Walter-Rittel, Sebastian Zschaeck

**Affiliations:** 1 Department of Nuclear Medicine, Charité-Universitätsmedizin Berlin, Berlin, Germany; 2 Department of Radiation Oncology, Charité-Universitätsmedizin Berlin, Berlin, Germany; 3 Research Center Dresden-Rossendorf, Dresden, Germany; 4 German Cancer Research Center, Heidelberg, Germany; 5 Department of Diagnostic and Interventional Radiology, Charité-Universitätsmedizin Berlin, Berlin, Germany; 6 Berlin Institute of Health (BIH), Berlin, Germany

**Keywords:** radiotherapy, head and neck squamous cell carcinoma, hypoxia, FMISO, positron emission tomography, FDG, PET

## Abstract

**Background:** Tumor hypoxia measured by dedicated tracers like [ ^18^F]fluoromisonidazole (FMISO) is a well-established prognostic factor in head and neck squamous cell carcinomas (HNSCC) treated with definitive chemoradiation (CRT). However, prevalence and characteristics of positron emission tomography (PET) measured hypoxia in patients with relapse after previous irradiation is missing. Here we report imaging findings of a prospective pilot study in HNSCC patients treated with re-irradiation.

**Methods:** In 8 patients with recurrent HNSCC, diagnosed at a median of 18 months after initial radiotherapy/CRT, [ ^18^F]fluorodeoxyglucose (FDG)-PET/CT (n=8) and FMISO-PET/MRI (n=7) or FMISO-PET/CT (n=1) were performed. Static FMISO-PET was performed after 180 min. MRI sequences in PET/MRI included diffusion-weighted imaging with apparent diffusion coefficient (ADC) values and contrast enhanced T1w imaging (StarVIBE). Lesions (primary tumor recurrence, 4; cervical lymph node, 1; both, 3) were delineated on FDG-PET and FMISO-PET data using a background-adapted threshold-based method. SUV _max_ and SUV _mean_ in FDG- and FMISO-PET were derived, as well as maximum tumor-to-muscle ratio (TMR _max_) and hypoxic volume with 1.6-fold muscle SUV _mean_ (HV _1.6_) in FMISO-PET. Intensity of lesional contrast enhancement was rated relative to contralateral normal tissue. Average ADC values were derived from a 2D region of interest in the tumor.

**Results:** In FMISO-PET, median TMR _max_ was 1.7 (range: 1.1-1.8). Median HV _1.6_ was 0.05 ml (range: 0-7.3 ml). Only in 2/8 patients, HV _1.6_ was ≥1.0 ml. In FDG-PET, median SUV _max_ was 9.3 (range: 5.0-20.1). On contrast enhanced imaging four lesions showed decreased and four lesions increased contrast enhancement compared to non-pathologic reference tissue. Median average ADC was 1,060 ×10 ^6^ mm ^2^/s (range: 840-1,400 ×10 ^6^ mm ^2^/s).

**Conclusions:** This pilot study implies that hypoxia detectable by FMISO-PET may not be as prevalent as expected among loco-regional recurrent, HPV negative HNSCC. ADC values were only mildly reduced, and contrast enhancement was variable. The results require confirmation in larger sample sizes.

## Introduction

Locally advanced head and neck squamous cell carcinomas (HNSCC) that are not associated with human papillomavirus (HPV) infections have an unfavorable prognosis. Standard treatment consists of definitive chemoradiation (CRT) with radiation doses of around 70 Gray (Gy). Acute toxicity is considerable and dose limiting in this approach. Nonetheless, about 50% of patients present with local or regional recurrence after definitive CRT ^1^. Re-irradiation is frequently chosen in cases with recurrence at the primary tumor site or regional lymph nodes. Due to radiation-induced long-term sequelae of the surrounding organs at risk, the re-irradiation dose is usually lower than in the primary setting. Consequently, tumors that are apparently radioresistant are treated with lower radiation doses than those applied in the primary situation. Not surprisingly, this approach is associated with a very unfavorable outcome. Especially in HPV negative tumors that are unresectable, re-irradiation is merely a palliative approach ^2^.

One important factor of radioresistance is tumor hypoxia. Hypoxia is associated with a more aggressive tumor phenotype as shown by various studies of different tumor entities ^3– 5^. Additionally, when treating patients with photon radiotherapy, hypoxia leads to a decreased cytotoxic effect of irradiation due to the lower availability of oxygen radicals. This effect can be specified by calculating the oxygen enhancement ratio (OER). Usually the OER is between two and four, which further underlines the important and strong effect of hypoxia in radiotherapy ^6^. One sophisticated method to measure hypoxia non-invasively is positron emission tomography (PET) with hypoxia specific radiotracers. The most commonly used hypoxia radiotracer is [ ^18^F]fluoromisonidazole (FMISO) ^7^. PET measured hypoxia provides a substantial and independent prognostic value in patients undergoing primary CRT for HNSCC ^8– 10^. Re-oxygenation, measured by repeated hypoxia PET during treatment, seems to be of even greater prognostic relevance in HNSCC with hardly any local tumor control in case of residual tumor hypoxia during the second week of CRT ^11– 14^. Given these data, we postulated that PET measured tumor hypoxia should also play an important role in local recurrent HNSCC after prior radiotherapy. To improve patient outcome by increasing re-oxygenation during CRT, a prospective pilot study was initiated to evaluate the effect of fever-range whole-body hyperthermia (FRWBH) on the tumor microenvironment in patients with re-irradiation for HNSCC. FRWBH has been shown to increase tumor perfusion in preclinical models ^15, 16^. The rationale of the GKH-TMM trial was to increase tumor perfusion and subsequently reduce tumor hypoxia by adding weekly FRWBH to re-irradiation. The primary endpoint of the trial was feasibility of FRWBH, which will be published separately when mature outcome data of patients are available. Secondary endpoints included changes of hypoxia and perfusion between pre-treatment and second week of fractionated CRT. Due to the lack of PET detectable hypoxia prior to treatment, we were not able to calculate these planned secondary outcome parameters. Here we report the imaging findings of the pretherapeutic PET and MRI scans within this trial. 

## Methods

The GKH-TMM, ARO-2018-3, study was registered at clinical trials (ClinicalTrials.gov identifier NCT03547388) and has been approved by the local Ethics committee (Charité Ethics committee, campus Virchow, EA2-047-18). All patients provided written informed consent to participate in the study and to publish results in a pseudonymized way.

The article complies with the reporting guidelines for observational studies (STROBE).

### Study design

The GKH-TMM study was a prospective Phase-I study to evaluate the effect of FRWBH on the tumor microenvironment. Inclusion criteria for this study were as follows: unresectable local, regional or loco-regional recurrent non HPV-associated HNSCC with prior high-dose radiotherapy of the head and neck region (either as definitive or as adjuvant CRT or radiotherapy), time interval between previous radiotherapy and recurrence between 6 months and 5 years, complete whole-body staging without evidence of distant metastases by [ ^18^F]fluorodesoxyglucose (FDG)-PET/CT, Eastern Cooperative Oncology Group (ECOG) performance status between zero and two, and age between 18 and 75 years. Prior definitive CRT was applied according to clinical guidelines with 32 fractions and simultaneous integrated boost (SIB) with 1.7 Gy single dose to elective nodal volume, 1.9 Gy to macroscopic lesions with enlarged safety margins and 2.2 Gy reduced safety margins. adjuvant CRT for high-risk patients was delivered in 30 fractions with SIB (1.8 Gy elective nodal treatment and 2.13 Gy to high-risk regions).

The study was designed as a pilot study with ten patients, who were recruited between April 2018 and March 2020. Eligible patients were asked to take part in the trial as a complementary method to routine treatment. The evaluation of tumor microenvironment was performed by pre-therapeutic FMISO-PET in combination with magnetic resonance imaging (MRI) with diffusion weighted imaging (DWI and ADC maps) and contrast enhanced high resolution imaging (post contrast T1w StarVIBE). FMISO-PET was scheduled prior to therapy and repeated at the end of the second week of CRT in case of evidence for pre-therapeutic hypoxia.

### Patients and treatment

Eight out of ten patients had pre-therapeutic FMISO-PET images. In two patients, FMISO-PET could not be performed due to logistical reasons. Out of the eight patients, seven patients underwent integrated PET/MRI, while one patient had to be examined by PET/CT due to severe claustrophobia.

All patients underwent hyperfractionated re-irradiation with two fractions of radiotherapy (1.2 Gy) per day and a minimum of eight hours between each fraction. Total treatment dose was 66 Gy, prescribed to the macroscopic tumor lesions plus 5 mm safety margin. Concomitant chemotherapy was not specified in the protocol but should preferentially include cisplatin due to its increased efficacy at mildly increased temperatures ^17^.

### FDG-PET/CT

Pretherapeutic FDG-PET/CT was performed with a dedicated PET/CT scanner (Philips Gemini TF 16, Philips, Amsterdam, The Netherlands). FDG-PET was performed to exclude distant metastases and to guide radiation treatment volume, additionally it was planned to analye geographic overlap between FDG and FMISO volumes. Patients were required to fast for at least 6h prior to tracer injection, and a blood glucose level ≤130 mg/dl was validated. After intravenous injection of a median of 259 MBq [ ^18^F]FDG (interquartile range [IQR], 252 to 296 MBq/kg; median, 4.4 MBq/kg; IQR, 3.9 to 4.9 MBq/kg), static PET acquisition was performed after 80 min (IQR, 68 to 84 min) for 2 or 3 min per bed position from base of skull to proximal femora in supine position (matrix, 144 × 144). PET data were reconstructed iteratively using ordered subset expectation maximization (OSEM; BLOB-OS-TF) with 3 iterations and 33 subsets and time of flight (voxel size, 4.0 × 4.0 × 4.0 mm ^3^) without resolution recovery (point spread function; PSF). Random correction, scatter correction and dead time correction were also included. Attenuation correction was performed based on a non-enhanced low-dose CT (slice thickness, 5 mm).

### FMISO-PET/MRI

Pretherapeutic FMISO-PET/MRI was performed with a dedicated PET/MRI scanner (Biograph mMR, Siemens Healthcare, Erlangen, Germany). After intravenous administration of 208 MBq (IQR, 185 to 212 MBq) [ ^18^F]FMISO (median, 3.5 MBq/kg; IQR, 3.0 to 3.7 MBq/kg), static PET images of the relapse location in the head and neck region were acquired after 180 min (IQR, 172 to 221 min) in one bed position with an acquisition time of 15 min (matrix, 172 × 172). PET data were reconstructed iteratively using OSEM with 3 iterations, 21 subsets and a 3 mm Gaussian in-plane filter (voxel size, 4.17 × 4.17 × 2.03 mm ^3^). Resolution recovery (point spread function) was not applied, and time of flight reconstruction was not available. Random correction, scatter correction and dead time correction were included. MR-based attenuation correction used the 3D CAIPIRINHA HiRes sequence (acceleration factor 5; voxel size, 1.3 × 1.3 × 3.0 mm ^3^). Approximately five minutes after intravenous injection of 0.2 ml/kg of gadoteric acid (Dotarem, Guerbet, Roissy CdG Cedex, France; 0.5 mmol/ml; 4 ml/s, followed by a 25 ml bolus of saline solution), contrast-enhanced imaging was performed by T1w 3D in-plane radial sampling by a fat-suppressed spoiled-Gradient Echo core sequence (StarVIBE; TR, 4.91 ms; TE, 2.14 ms; flip angle (FA), 12°; field of view [FOV], 220 × 220 mm ^2^; voxel size, 0.7 × 0.7 × 1.3 mm ^3^). Diffusion-weighted imaging and apparent diffusion coefficient (ADC) maps of the tumor volume were acquired with a fat-saturated multi-shot readout-segmented echo-planar sequence (TR, 3840 ms; TE1, 61 ms; TE2, 100 ms; b1, 50 s/mm ^2^; b2, 800 s/mm ^2^; EPI factor 82; FA, 180°; FOV, 220 × 220 mm ^2^; voxel size, 1.1 × 1.1 × 4.0 mm ^3^); a pre- and post-contrast T1w Turbo Spin Echo (TSE) sequence (TR 692 ms; TE, 9 ms; FA, 142°; FOV, 180 × 180 mm ^2^; voxel size, 0.6 × 0.6 × 3.0 mm ^3^) and a T2w turbo -inversion recovery magnitude (TIRM) sequence (TR, 4510 ms; TE, 40 ms; FA, 140°; FOV, 200 × 200 mm ^2^; voxel size, 0.6 × 0.6 × 3.0 mm ^3^) were acquired. Parallel imaging was enabled by Generalized Autocalibrating Partially Parallel Acquisition (GRAPPA).

### FMISO-PET/CT

In a single patient with severe claustrophobia, pretherapeutic FMISO-PET/CT was performed instead of PET/MRI. After intravenous injection of 171 MBq [ ^18^F]FMISO (1.8 MBq/kg), PET acquisition was performed with the above-named PET/CT scanner (Philips Gemini TF 16) after 197 min for 15 min in a single bed position (matrix, 144 × 144). PET data were reconstructed with identical parameters as described for FDG-PET/CT imaging. Additionally, a venous-phase contrast enhanced, diagnostic CT (automated tube current modulation; maximum tube current-time product, 200 mAs; tube voltage, 120 kV; FOV, 395 × 395 mm ^2^; voxel size, 0.77 × 0.77 × 3.0 mm ^3^) was performed 100 s after intravenous injection of 120 ml of Imeron 350 (bolus rate, 2 ml/s; Bracco Imaging Deutschland GmbH, Konstanz, Germany).

### Image evaluation

Image analysis was performed as previously published and as shortly described in the following passage using ROVER software (version 3.0.50h; ABX, Radeberg, Germany; available freely for research purposes on request) without any preprocessing of the final image data ^18^. After co-registration of diagnostic FDG-PET/CT images and FMISO-PET/MRI or PET/CT images by the mutual information algorithm, correct alignment of the primary tumor volume was verified, and co-registration was corrected, if deemed necessary. The tumor (primary tumor recurrence or lymph node) was semi-automatically delineated in the PET images using a background-adapted threshold-based algorithm ^19, 20^. Central necrotic tumor areas or areas that were suspicious of tumor in MRI or CT were subsequently included manually to the primary tumor/lymph node volume. A reference region of interest (ROI) was delineated in the deep neck muscles contralateral to the primary tumor with a spheroid of 16 mm diameter. The ROIs of the tumor and of the muscle were transferred to the hypoxia PET for calculation of the maximum standardized uptake value (SUV _max_; normalized to the body weight) of the tumor, of the tumor-to-muscle ratio (TMR) and for thresholding of hypoxic volumes, e.g. the commonly used hypoxic volume with uptake above 1.6 times of the average muscle uptake (HV _1.6_).

The intensity of lesional contrast enhancement was rated by an experienced radiologist (9 years of experience) relative to the corresponding contralateral normal tissue using either the contrast-enhanced T1w images (StarVIBE sequence; n=7 patients) or the contrast-enhanced CT data (n=1 patient). Intensity was rated on a three point Likert-type item as “less intense”, “comparable intensity” or “more intense” compared to the contralateral reference tissue. Mean ADC values of the tumor lesions were measured by the same radiologist using representative 2D region of interest placed in the tumor volume.

### Statistical analysis

Statistical analysis was performed using SPSS (version 26, IBM, Armonk, NY, USA). On the basis of the small sample size, non-normal data distribution was assumed, and descriptive data were expressed as median, IQR and range, unless otherwise specified.

## Results

Eight patients with available pretherapeutic FMISO-PET imaging are reported here (PET/MRI, n=7; PET/CT, n=1). Characteristics of patients and treatment are summarized in Table 1.

**Table 1.** Patient and treatment characteristics. abbreviations: OC = oral cavity, OP = oropharynx, HP = hypopharynx, def CRT = definitive chemoradiation, adj CRT = adjuvant chemoradiation, T = local recurrence, N = regional recurrence.

Patient Number	Age	Gender	Primary tumor location	Primary UICC stage	Primary treatment	Site of recurrence	Time to recurrence (months)	Concomitant systemic therapy
#1	60	male	OC	IVA	def CRT (70.4 Gy)	T	34	cisplatin
#2	69	male	Larynx	IVA	adj CRT (63.9 Gy)	T	6	cetuximab
#3	55	male	OP	IVA	def CRT (70.4 Gy)	T + N	12	nivolumab
#4	60	male	OP	IVA	def CRT (70.4 Gy)	T	25	cisplatin
#5	60	male	HP	IVA	def CRT (70.4 Gy)	T + N	58	cisplatin
#6	67	female	OP	IVA	adj CRT (63.9 Gy)	T + N	38	cisplatin
#7	55	male	OP	IVA	def CRT (70.4 Gy)	T	9	cisplatin
#8	57	male	OC	IVB	adj CRT (63.9 Gy)	N	8	cisplatin

### Hypoxia in FMISO-PET

Assessment of tumor hypoxia in static FMISO-PET images three hours post injection revealed absence of detectable hypoxia in almost all patients. The median FMISO TMR _max_ was 1.7 (IQR, 1.3 to 1.8; range, 1.1 to 1.8). Only two patients showed hypoxic volumes that were delineable using the 1.6-fold average background muscle activity threshold (HV _1.6_). The median HV _1.6_ of all 8 patients was 0.05 ml (IQR, 0 to 0.33 ml; range, 0 to 7.3 ml). Supplementary figure 1 and 2 show both patients with detectable HV _1.6_ volumes and corresponding FDG PET CTs. As can bee seen, patient #4 shows scattered FMISO accumulation within and around the macroscopic lesion and only patient #6 shows a relatively well defined hypoxic volume within the macroscopic lesion. No lesion showed a TMR _max_ >2.0. Notably, especially the largest recurrent lesions did not show any detectable hypoxia (HV _1.6_ = 0 ml).

### Uptake in FDG-PET

All lesions showed unequivocal and extensive FDG uptake (median MTV, 11.2 ml; IQR, 6.0 to 16.8 ml; range, 1.0 to 51.7 ml). The median SUV _max_ was 9.3 (IQR, 8.4 to 13.6; range, 5.0 to 20.1), and the median SUV _mean_ was 6.3 (IQR, 5.8 to 8.6; range, 3.0 to 12.6).

**Figure 1.**
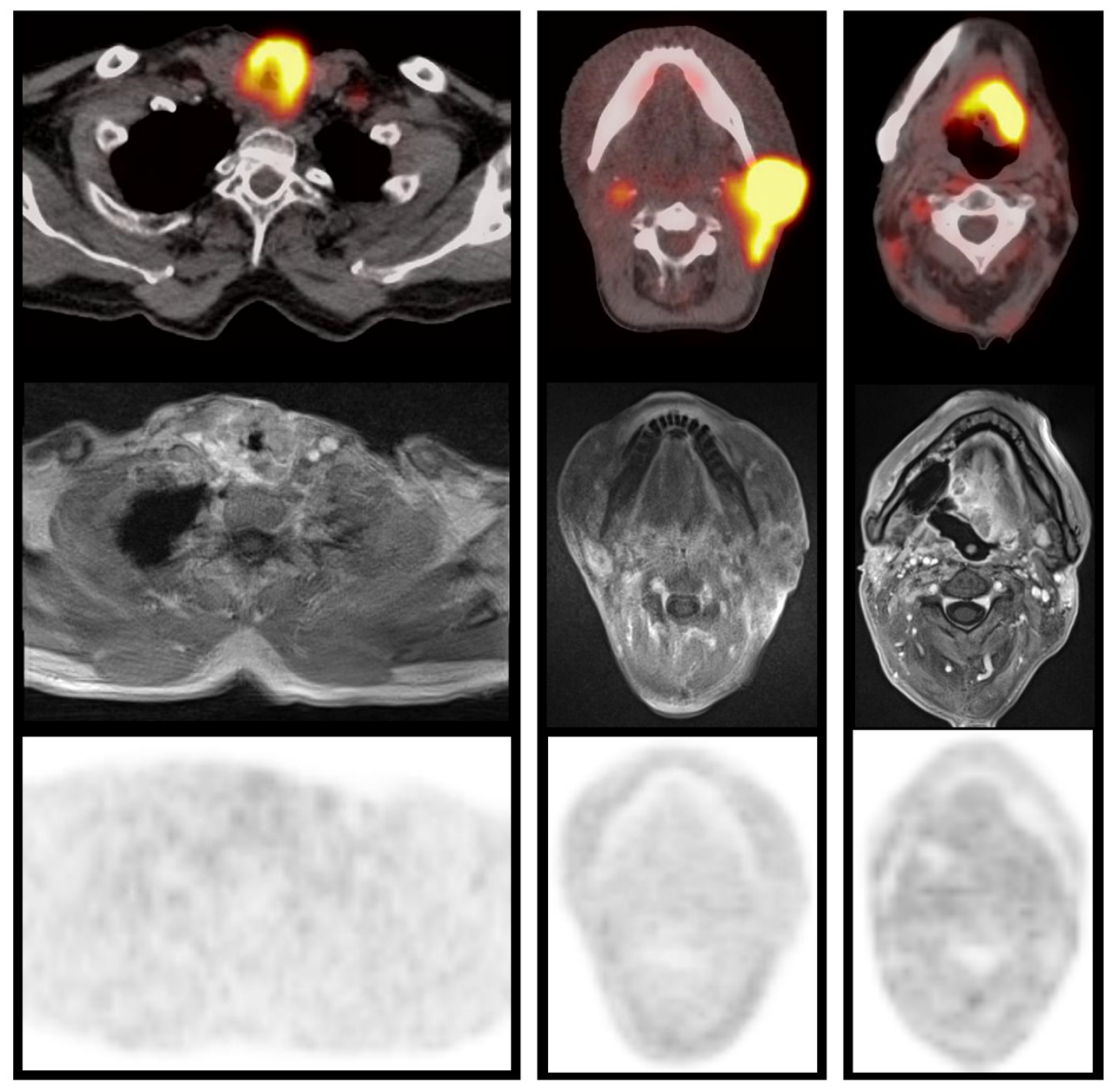
Patient examples. Examples of patients with local recurrence of a larynx cancer (left; patient #2), cervical lymph node metastasis (middle; patient #3) or oropharyngeal cancer relapse (right; patient #4), respectively. Fused FDG-PET/CT above, corresponding contrast-enhanced T1w MRI (StarVIBE) in the middle, and FMISO-PET below. In each patient, FDG uptake of the recurrent lesion is extensive and high, while specific FMISO uptake was absent (HV ^1.6^ ≤1.0 ml).

Details of FDG-PET and FMISO-PET parameters for all patients are shown in Table 2. Figure 1 shows exemplary FDG-PET/CT scans and FMISO-PET/MRI scans.

**Table 2.** FDG-PET, FMISO-PET and MRI parameters. Abbreviations: MTV = metabolic tumor volume, SUV _max_ = maximum standardized uptake value, SUV _mean_ = mean standardized uptake value, TMR _max_ = maximum tumor-to-muscle ratio, HV ^1.6 ^= hypoxic volume with a threshold of 1.6 times the average background/ muscle uptake; ADC = apparent diffusion coefficient.

Patient Number	FDG MTV (ml)	FDG SUV _max_	FDG SUV _mean_	FMISO SUV _max_	FMISO SUV _mean_	FMISO TMR _max_	FMISO HV ^1.6^ (ml)	Contrast enhancement relative to the reference	Average ADC value (10 ^6^ mm ^2^/s)
#1	0.95	5.9	5.1	1.0	0.6	1.8	0.1 ^*^	more intense	1,357
#2	31.3	18.3	11.5	1.3	0.9	1.59	0	less intense	1,050
#3	51.7	20.1	12.6	0.7	0.4	1.12	0	less intense	1,000
#4	10.7	12.1	7.6	1.0	0.8	1.79	1.0	more intense (periphery)	1,400
#5	2.5	9.3	6.0	0.8	0.7	1.18	0	more intense (periphery)	1,060
#6	12.0	9.3	6.4	2.0	1.6	1.84	7.3	more intense	*n.a.* (CT only)
#7	11.6	9.3	6.2	0.9	0.6	1.79	0.1 ^*^	less intense	840
#8	7.2	5.0	3.0	0.6	0.4	1.4	0	less intense	1,065

*Only a single voxel above the threshold

### Contrast enhancement and ADC values

Tumor contrast enhancement was less intense compared to the contralateral reference in four of eight patients (lymph node only, n=1; primary tumor recurrence ± lymph node, n=3; Table 2). In the remaining four patients, enhancement was more intense, at least in parts of the lesion, compared to the reference tissue (primary tumor ± lymph node, n=4).

The median average ADC value was 1,060 × 10 ^6^ mm ^2^/s (IQR, 1,025 to 1,211 × 10 ^6^ mm ^2^/s; range, 840 to 1,400 × 10 ^6^ mm ^2^/s).

## Discussion

Here we report pretrial data from the first trial that evaluated tumor hypoxia by specific hypoxia PET in previously irradiated, recurrent HNSCC. Data on hypoxia measured by PET tracers in the recurrent disease situation after radiotherapy is absent. To the best of our knowledge, there is only one study that investigated various tumor locations and histologies with hypoxia PET. In the mentioned study, the authors describe one patient with recurrent adenocarcinoma of the uterus who showed a relatively high uptake of FMISO. It is not clear if this patient was previously treated with radiotherapy/CRT or presented recurrence after initial surgery ^21, 22^.

Very surprisingly, the incidence of hypoxia, determined by FMISO-PET, was extremely low in our study investigating recurrent HNSCC. The largest analysis of hypoxia PET images from five European centers established the hypoxia parameter TMR _max_ 2.0 as a suitable value to distinguish high- and low-risk treatment-naive patients. The prevalence of hypoxia (defined as TMR _max_ > 2.0) was relatively high in primary tumors. PET hypoxia was found in 48% of all patients; among those who received FMISO-PET, 61% showed hypoxic tumors/lesions ^18^. In the present trial of previously irradiated HNSCC, none of the eight included patients showed hypoxic tumors according to these cut-off values, despite the fact that patients with relatively large recurrent lesions were included (see Table 2). This finding may indicate that microenvironmental mechanisms of tumor radioresistance might be very different between treatment-naïve and previously irradiated tumors. This also seems to be the case with genetic alterations ^23, 24^. Dose painting for re-irradiation does not seem feasible in FMISO-PET due to the low tracer uptake, i.e. low rates of hypoxia in this patient collective [ 25, 26,p.33].

In addition to late static FMISO-PET images, dynamic acquisition from 0 to 40 min post injection has been proposed. Combining dynamic and static PET images for a voxel-wise 2-compartment model of [ ^18^F]FMISO accumulation, Thorwarth *et al.* were able to identify patients with treatment-naïve HNSCC with either favorable or very poor local tumor control after radiotherapy. The derived dynamic parameter *M _FMISO_* was superior to TMR _max_ in predicting local tumor control ^27^. Dynamic FMISO-PET acquisition was not available in our patient collective. However, it is questionable if dynamic data would provide any added value in lesions that are negative on late FMISO images.

In addition to mostly absent hypoxia, ADC values were relatively high in all tumors with a median of >1,000 × 10 ^6^ mm ^2^/s, which corresponds to only mildly restricted diffusion. Only in one tumor (patient #7), the average ADC was below 1,000 × 10 ^6^ mm ^2^/s. These MRI findings are consistent with the literature ^28– 30^. Hwang *et al.* reported average ADC values of 1,200 × 10 ^6^ mm ^2^/s in patients with recurrent HNSCC after initial treatment compared to an average of 1,650 × 10 ^6^ mm ^2^/s in lesions corresponding to post-therapeutic changes ^30^. Previous reports on the association between tissue hypoxia in different tumor entities and properties in DWI or corresponding ADC maps are inconsistent. Hino-Shishikura *et al.* reported a negative correlation between ADC values and the SUV _max_ and tumor-to-background ratios in brain tumors obtained with hypoxia-specific PET imaging ^31^. Hompland *et al.* demonstrated a weak negative correlation between ADC values and hypoxic fraction assessed by pimonidazole tissue staining in malignant melanoma xenografts. Moreover, ADC values as high as 1,000 × 10 ^6^ mm ^2^/s were only observed in tumors with hypoxic volumes <10% of the total tumor volume ^32^. In contrast, a positive correlation was reported in murine melanoma tumors (but imaging was performed *ex vivo*) ^33^. Swartz *et al.* and Carmona-Bozo *et al.* found no correlation between hypoxia and ADC values in oropharyngeal and breast cancer lesions, respectively ^34, 35^.

Contrast enhancement (StarVIBE MRI sequence or contrast-enhanced CT) was atypically low in 4/8 lesions, and hypoxia was also absent in these tumors ( Table 2). Among the 4/8 lesions with substantial contrast enhancement (at least in the tumor periphery), some showed small hypoxic proportions as determined by the FMISO HV _1.6_. Gerstner *et al.* found that high lesional cerebral blood flow in glioblastoma quantified by dynamic susceptibility contrast MRI correlated positively with the hypoxic volume in FMISO-PET. The authors postulated that abnormal tumor vasculature contributes to hypoxia ^36^. This may explain the lack of substantial and extensive contrast enhancement in the current mostly non-hypoxic tumors. However, the current sample size was too small to conclude if there is a negative correlation between contrast enhancement and tumor hypoxia. Dynamic contrast MRI sequences were not evaluated in this study, and contrast kinetics – as described by 36 – could not be assessed.

In summary, the current pilot study implies that relevant hypoxia, which is detectable by static FMISO-PET, may not be as prevalent among recurrent lesions of HPV negative HNSCC as expected. However, studies with substantially larger sample sizes would be required for a definite statement or more differentiated analyses (e.g., dependence of hypoxia on the type and localization of the recurrent lesion). This is also true for the investigation of a possible association of low tumor hypoxia with high ADC values and low contrast enhancement in these recurrent lesions. However, recruitement of HPV negative recurrent HNSCC into time consuming imaging trials remains challenging. Patient compliance is often low and comorbidities can hamper acquisition of sophisticated PET/ MRI protocols.

## Data availability

All data underlying the results are available as part of the article (concerning absolute values of imaging parameters). Original imaging data cannot be made publicly available due to concerns regarding data protection since the imaging data contained several high-resolution methods with unique personal properties. German data protection is not only restricted by Ethics committees, but also by dedicated data protection agencies. One general concern of German data protection agencies, including Charité data protection commission, is that image information that is uploaded to public available repositories might be accessed by countries that do not comply with current data protection agreements. Therefore, pseudonymized data can be shared upon reasonable request by contacting the corresponding author. Access to the data will be granted after ethical approval and after ensuring that adequate data protection is adhered by the country of the applicant.

### Extended data

Figshare: Supplementary figures with FDG and FMISO PET images of patients #4 and #6

https://doi.org/10.6084/m9.figshare.14170061.v3 37

This project contains the following underlying data:

- Supplementary Figure 1. (Patient #4 with FDG PET (first row) and fused FDG-PET CT (second row) and hypoxia FMISO PET (third row) with fused MRI (last row). The FDG volume is automatically segmented within the yellow sphere. Applying the 1.6 muscle threshold within this sphere leads to scattered hypoxic volumes without clear relation to the macroscopic tumor.)
- Supplementary Figure 2. (Patient #6 with FDG-PET (first row), fused CT image (second row). FMISO PET (third row) and FMISO-PET CT (last row). This time the 1.6 hypoxic threshold shows a hypoxic volume within the macroscopic tumor and only little extra-tumoral uptake in the surroundings.)

Data are available under the terms of the Creative Commons Zero “No rights reserved” data waiver (CC0 1.0 Public domain dedication).
