# Supplementary material for: PET measured hypoxia and MRI parameters in re-irradiated head and neck squamous cell carcinomas: findings of a prospective pilot study
Source: F1000Res. 2021 Mar 16;9:1350. Originally published 2020 Nov 19. [Version 2] doi: 10.12688/f1000research.27303.2 (PMC7970429; doi:10.12688/f1000research.27303.2)
Supplement: Peer review history

# Peer review: Reviewer response for version 2

**DOI:** 10.5256/f1000research.55191.r81544

**Published:** 2021-03-17

**Authors:** Maria Picchio

**Affiliations:** 1 Unit of Nuclear Medicine, IRCCS San Raffaele Scientific Institute, Milan, Italy; 2 Vita-Salute San Raffaele University, Milan, Italy

In the revised form of the manuscript the limitations of the very limited and heterogeneous population are still present. However, an explanation has been provided for all the points underlined by the reviewers. I have no further comments to make.

Is the work clearly and accurately presented and does it cite the current literature?

Yes

If applicable, is the statistical analysis and its interpretation appropriate?

Yes

Are all the source data underlying the results available to ensure full reproducibility?

Yes

Is the study design appropriate and is the work technically sound?

Yes

Are the conclusions drawn adequately supported by the results?

Yes

Are sufficient details of methods and analysis provided to allow replication by others?

Yes

Reviewer Expertise:

Molecular Imaging in oncology

I confirm that I have read this submission and believe that I have an appropriate level of expertise to confirm that it is of an acceptable scientific standard.

# Peer review: Reviewer response for version 1

**DOI:** 10.5256/f1000research.30169.r79089

**Published:** 2021-03-01

**Authors:** Maria Picchio

**Affiliations:** 1 Unit of Nuclear Medicine, IRCCS San Raffaele Scientific Institute, Milan, Italy; 2 Vita-Salute San Raffaele University, Milan, Italy

The present manuscript deals with a very interesting issue reporting preliminary data on the use of a PET radiotracer, 18F-FMISO, to measure hypoxia in head and neck squamous cell carcinoma patients with relapse after previous irradiation. Eight patients have been included, 7/8 studied by FMISO PET/MRI and 1/8 by using PET/CT.

 As for strengths, the paper is very well written and methodologically correct.

 In addition, although 18F-FMISO is a well-established prognostic factor in head and neck squamous cell carcinomas treated with definitive chemoradiation, data on prevalence and characteristics of PET measured hypoxia in patients with relapse after previous irradiation are still missing. This paper report imaging findings of pretherapeutic PET and MRI scans within a prospective trial.

 As weakness, the number of patients included is very limited and the population is heterogeneous. In addition, two different scanners have been used (7 PET/MRI and 1 PET/CT). The rationale and the use of FDG in this preliminary evaluation has not been definitely defined and commented.

 It should be also clarified why 1.6-fold muscle has been considered to define hypoxic volume. In literature other values have been also reported (i.e. 1.2; 1.4)

Is the work clearly and accurately presented and does it cite the current literature?

Yes

If applicable, is the statistical analysis and its interpretation appropriate?

Yes

Are all the source data underlying the results available to ensure full reproducibility?

Yes

Is the study design appropriate and is the work technically sound?

Yes

Are the conclusions drawn adequately supported by the results?

Yes

Are sufficient details of methods and analysis provided to allow replication by others?

Yes

Reviewer Expertise:

Molecular Imaging in oncology

I confirm that I have read this submission and believe that I have an appropriate level of expertise to confirm that it is of an acceptable scientific standard, however I have significant reservations, as outlined above.

# Response

**Published:** 2021-03-03

**Authors:** Sebastian Zschaeck

**Affiliations:** Charite - Medical University Berlin, Germany

We thank the reviewer for her helpful and positive comments

*As weakness, the number of patients included is very limited and the population is heterogeneous. In addition, two different scanners have been used (7 PET/MRI and 1 PET/CT). The rationale and the use of FDG in this preliminary evaluation has not been definitely defined and commented.*

 We agree that the number of patients is limited and somehow heterogenous (especially regarding surgery). However, one has to bear in mind the low compliance of this group of HPV negative HNSCC and local relapse. Therefore it was already relatively difficult to recuit these patients into a trial with time consuming additional imaging - as a side note, only 1/3 of all screened patients could be recruited to this trial. Usually the same PET-MRI scaner was used, however due to severe claustrophobia one patient was scanned on the PET-CT.

 FDG is routinely used for re-irradiation in our department for staging and better target delineation. Additionally FDG PET should be used to define regions of FDG and hypoxia overlap within the macroscopic recurrent tumor. We added these information accordingly.

*It should be also clarified why 1.6-fold muscle has been considered to define hypoxic volume. In literature other values have been also reported (i.e. 1.2; 1.4)*

 We fully agree that other thresholds have been reported. The 1.6 fold score is taken from the current meta-analysis. However, even this relatively high threshold led to scattered hypoxia volumes inside and outside the macroscopic tumor lesions. For better ilustration we included images of the two patients with (small) HV1.6 volumes inside the macrospopic tumor (#4 and #6) as supplementary information and hope that this visualizes that a true hypoxic volume can probably only defined in patient #6.

# Peer review: Reviewer response for version 1

**DOI:** 10.5256/f1000research.30169.r75138

**Published:** 2020-12-02

**Authors:** Jacqueline Kelly

**Affiliations:** 1 Department of Therapeutic Radiology, Yale School of Medicine, New Haven, CT, USA

In this manuscript, the authors explore the novel and important question of hypoxia levels in recurrent HNSCC that has previously been irradiated with high-dose RT. Because hypoxia has been shown to be prognostic in the definitive HNSCC RT setting, and as re-RT is toxic and may be effective in only very select patient populations, the knowledge gained from this study could be used to select appropriate candidates who may be more likely to have a durable response to re-RT. Surprisingly, the incidence of hypoxia was extremely low in the present study, albeit in a very limited number of patients in a heterogeneous patient population. 

 The paper is well-written, with clear methodology, presentation of the results, and an appropriate discussion. In viewing Table 1, it appears initial treatment was uniform, with def CRT patients receiving 70.4 Gy and adj CRT receiving 63.9. Since these doses are clearly not achieved using the commonly used 2Gy/fraction, it may be worth mentioning the fractionation schedule used with the initial treatment course. Altered fractionation schedules have at least theoretical advantages relative to OER effects and thus this should simply be noted.  

 I would also recommend in the conclusion reiterating that these findings apply to only the HPV(-) HNSCC population. 

 Overall, a soundly conducted study, unfortunately with conclusions limited by a very low number of patients.

Is the work clearly and accurately presented and does it cite the current literature?

Yes

If applicable, is the statistical analysis and its interpretation appropriate?

Yes

Are all the source data underlying the results available to ensure full reproducibility?

Yes

Is the study design appropriate and is the work technically sound?

Yes

Are the conclusions drawn adequately supported by the results?

Yes

Are sufficient details of methods and analysis provided to allow replication by others?

Yes

Reviewer Expertise:

Radiation therapy, radiation biology

I confirm that I have read this submission and believe that I have an appropriate level of expertise to confirm that it is of an acceptable scientific standard.

# Response

**Published:** 2021-03-03

**Authors:** Sebastian Zschaeck

**Affiliations:** Charite - Medical University Berlin, Germany

We thank the reviewer for her useful comments.

 We included a description of the initial treatment schedule (definitive and adjuvant CRT). Both regimes are applied according to clinical guidelines, using simultaenous integrated boost. However, the slight increase of single dose (2.13 or 2.2 Gray) is probably within the range that can be regarded normo-fractionated (1.8 to 2.0 or 2.2 Gray, according to definition), therefore we do not expect a major impact on OER.

 We added the limitation that these finding apply only to HPV negative HNSCC in the abstract and in the results section.
